# A flow cytometry-based workflow for detection and quantification of anti-plasmodial antibodies in vaccinated and naturally exposed individuals

**DOI:** 10.1186/1475-2875-11-367

**Published:** 2012-11-06

**Authors:** Anthony Ajua, Thomas Engleitner, Meral Esen, Michael Theisen, Saadou Issifou, Benjamin Mordmüller

**Affiliations:** 1 Institute of Tropical Medicine, University of Tübingen, Wilhelmstraße 27, Tübingen D-72074, Germany; 2 Centre de Recherche Médicale de Lambaréné (CERMEL), Lambaréné, BP 118, Gabon; 3 Center for Medical Parasitology at Department of International Health, Immunology and Microbiology, University of Copenhagen, Bartholinsgade 2, Copenhagen K, 1356, Denmark; 4 Department of Clinical Biochemistry and Immunology, Statens Serum Institut, Artillerivej 5, Copenhagen S, 2300, Denmark

**Keywords:** Malaria, Flow cytometry-based IFA, Algorithmic data analysis, Anti-malarial antibodies, Human serum

## Abstract

**Background:**

Antibodies play a central role in naturally acquired immunity against *Plasmodium falciparum*. Current assays to detect anti-plasmodial antibodies against native antigens within their cellular context are prone to bias and cannot be automated, although they provide important information about natural exposure and vaccine immunogenicity. A novel, cytometry-based workflow for quantitative detection of anti-plasmodial antibodies in human serum is presented.

**Methods:**

Fixed red blood cells (RBCs), infected with late stages of *P*. *falciparum* were utilized to detect malaria-specific antibodies by flow cytometry with subsequent automated data analysis. Available methods for data-driven analysis of cytometry data were assessed and a new overlap subtraction algorithm (OSA) based on open source software was developed. The complete workflow was evaluated using sera from two GMZ2 malaria vaccine trials in semi-immune adults and pre-school children residing in a malaria endemic area.

**Results:**

Fixation, permeabilization, and staining of infected RBCs were adapted for best operation in flow cytometry. As asexual blood-stage vaccine candidates are designed to induce antibody patterns similar to those in semi-immune adults, serial dilutions of sera from heavily exposed individuals were compared to naïve controls to determine optimal antibody dilutions. To eliminate investigator effects introduced by manual gating, a non-biased algorithm (OSA) for data-driven gating was developed. OSA-derived results correlated well with those obtained by manual gating (r between 0.79 and 0.99) and outperformed other model-driven gating methods. Bland-Altman plots confirmed the agreement of manual gating and OSA-derived results. A 1.33-fold increase (p=0.003) in the number of positive cells after vaccination in a subgroup of pre-school children vaccinated with 100 μg GMZ2 was present and in vaccinated adults from the same region we measured a baseline-corrected 1.23-fold, vaccine-induced increase in mean fluorescence intensity of positive cells (p=0.03).

**Conclusions:**

The current workflow advances detection and quantification of anti-plasmodial antibodies through improvement of a bias-prone, low-throughput to an unbiased, semi-automated, scalable method. In conclusion, this work presents a novel method for immunofluorescence assays in malaria research.

## Background

Malaria is a major cause of morbidity and mortality in endemic countries with African children carrying the major burden of the disease. An efficacious malaria vaccine would be a cost-effective and easy-to-implement intervention to complement current control strategies, but until today no malaria vaccine is registered for routine use [1], although one product – RTS,S/AS01 – has shown promising results in a clinical phase III study [2]. In contrast to vaccines containing pre-erythrocytic antigens, such as RTS,S, vaccines directed against the asexual blood stage are thought to act mainly through antibodies (Abs). Hence, it is hypothesized that anti-plasmodial Ab concentrations similar to those acquired upon natural exposure are required to attain semi-immunity, a type of non-sterile but robust immunity that protects from clinical complications and excessive parasite replication [1,3]. The main evidence for the role of Abs in semi-immunity comes from studies where purified Abs from African malaria-immune adults were successfully used to treat non-immune malaria patients [4,5] within Africa or, as an extension of this, in South-East Asia [5]. The mechanisms, properties, and specificities of Abs that mediate protection in malaria, however, remain unknown [3].

During clinical development of the malaria vaccine candidate GMZ2 [6-8], it was noted that current assays to monitor immunogenicity and pre-existing immunity to malaria with intact parasites are bias-prone and difficult to standardize. Conventionally, most approaches are based on enzyme-linked immunosorbent assay (ELISA) using recombinant proteins or synthetic peptides as bait antigen [9]. These could differ from their corresponding native parasite counter-parts in their folding and post-translational modifications, potentially altering the target protein’s antigenic properties [3]. In addition, the degree of parasite antigen exposure to the immune system (e.g. the effects of localization in protein complexes or organelles) may be crucial for an effective anti-parasitic reaction or as a correlate for successful vaccination. This becomes even more important as second-generation, multi-subunit and whole cell vaccines enter clinical development [10]. As such, the use of microscopic immunofluorescent antibody assay (IFA) to study Ab concentrations against total parasite proteins expressed in mature blood stage schizonts and merozoites using native parasites [9,11] may provide important insights into the Ab-mediated anti-plasmodial immune response.

Microscopic IFA however, has many setbacks; quantification is done by determination of titers and quality control remains problematic due to poor assay standardization and potential investigator bias. Additionally, the assay is not scalable and, therefore, investigation of larger cohorts proves prohibitive [12]. On the other side, in skillful hands, microscopic IFA is highly sensitive and specific and provides information about the ability of vaccine-induced Abs to bind to native parasite molecules [9]. This being known, a scalable, sensitive, reproducible, and quantitative assay based on flow cytometry, a well-established and automatable technology, which is widely available in developing countries [13], was proposed to improve microscopy-based assays and allow for high throughput measurements [14-16]. A major drawback of this approach is that flow cytometry data are routinely analysed by manual gating, which is potentially biased and inconsistent [15]. To overcome these challenges, a data-driven algorithm was developed to automatically analyse flow cytometric data and a novel workflow for a medium-throughput, sensitive, and reliable flow cytometry-based immunoassay for the detection and quantification of anti-plasmodial antibodies in human serum is presented.

## Methods

### Study populations and serum samples

Serum samples from Day 0 (before vaccination) and Day 84 (4 weeks after the last of three vaccine administrations) were collected from two clinical trials of GMZ2. Details of the volunteers and vaccination schedules are described elsewhere [7,8]. In brief, two double-blind, randomized phase Ib clinical trials of GMZ2 were performed in Lambaréné, Gabon; one enrolled adults [8], the other pre-school children [7]. The trial involving healthy Gabonese adults took place between July 2007 and August 2008. Twenty participants received 100 μg GMZ2 adjuvanted with aluminium hydroxide (alum) subcutaneously on Days 0, 28 and 56, whereas the 20 participants in the control group received rabies vaccine intramuscularly at the same time points (Days 0, 28, and 56). The pediatric trial took place from September 2008 to October 2009 and involved 30 healthy pre-school children aged 1 to 5 years. The children received three doses of either rabies control vaccine (n = 10), 30 μg GMZ2 (n = 10) or 100 μg GMZ2 (n = 10). The 3 doses were administered one month apart (Days 0, 28 and 56) by intramuscular injection.

Both studies were reviewed by the regional ethics committee (*Comité d*‘*Ethique Régional Indépendant de Lambaréné*; CERIL) and followed Good Clinical Practice guidelines as defined by the International Conference on Harmonization. All studies were conducted according to the principles of the Declaration of Helsinki in its 5^th^ revision.

### *Plasmodium falciparum* culture, synchronization and enrichment for late stages

The laboratory-adapted *P*. *falciparum* strain 3D7A, obtained from the Malaria Research and Reference Reagent Resource (ATCC, Virginia, USA) was cultured in complete medium (RPMI 1640, 25 mM HEPES, 2.4 mM L-glutamine, 50 μg/mL gentamicin and 0.5% w/v Albumax). Confirmatory experiments were done using the *P*. *falciparum* strain Dd2 obtained from the same source. All cultures were maintained at 37°C in an atmosphere of 5% CO_2_ and 5% O_2_, with daily changes of medium at 5% haematocrit and dilution with red blood cells when the parasitaemia exceeded 5%.

Parasite cultures were synchronized at early ring stage by treatment with 5% D-sorbitol (Sigma, St. Louis, USA) for 10 min at 37°C. Isolation of synchronized *P*. *falciparum* parasites (late trophozoite and schizont) was performed using LD-MACS magnetic columns (Miltenyi Biotec, Gladbach, Germany), as described previously, at a parasitaemia of about 5% [17]. Following enrichment, the purity of the parasite preparation was verified by light microscopy and by flow cytometry after DNA staining with Hoechst 33342. In later experiments, Vybrant DyeCycle violet stain (Invitrogen, Germany) replaced Hoechst 33342.

### Flow cytometry-based immunofluorescence assay to detect anti-plasmodial antibodies

Preparation of parasites for cytometry was based on a previously described fixation protocol [18]. Briefly, *P*. *falciparum* culture enriched for late developmental parasite stages were washed once in phosphate buffered saline (PBS) and fixed by incubation in a combination of PBS with 4% EM grade paraformaldehyde (Merck, Germany) and 0.0075% EM grade glutaraldehyde (Sigma-Aldrich, Germany) for 30 min. Fixed cells were washed again in PBS and permeabilized for 10 min in PBS/0.1% Triton-X-100 (TX100) (Sigma-Aldrich, Germany). After another PBS wash step, free aldehyde groups were reduced by incubating cells for 10 min in PBS with 0.1 mg/ml sodium borohydride (Merck, Germany). The preparation was washed again with PBS and cells blocked in PBS/3% BSA. The cells were counted using a haemocytometer (Neubauer–counting chamber) and the pellet reconstituted in PBS to standardize the number of cells used in the assay. As a modification of the original protocol, all subsequent handling of cells in 1.5 ml sample tubes (Eppendorf, Hamburg, Germany) was performed in 96-well round-bottom plates (Corning, NY, USA) instead. To detect parasite-specific immunoglobulin G (IgG), parasite suspension (2 μl of approx. 5.0 x 10^7^ cells per ml) was added into each well of the 96-well plate resulting in a total volume of 100 μl of test sera and control samples (each diluted in PBS/3%BSA) and allowed to bind for 1 h at RT on a plate shaker. After incubation, the cells were washed thrice with 150 μl of PBS to remove excess unbound primary antibody. Subsequently, pellets were resuspended in 100 μl AlexaFluor 488 goat anti-human IgG (Molecular Probes, Germany), diluted in PBS/3%BSA, and incubated in the dark for 1 hour. Following three washes with PBS, cells were stored at 4°C in the dark prior to cytometric analysis.

Antibody dilutions of both primary and secondary antibodies used in the assay were pre-determined through checkerboard titration experiments. The combination of antibody dilutions that gave the best separation between negative and positive fluorescent parasites was selected and used in subsequent experiments. Furthermore, different dilutions of three second-step AlexaFluor-conjugated goat anti-human IgG antibodies as well as a non-conjugated anti-histidine rich protein 2 (HRP2) monoclonal IgM (used as positive control) were tested. In addition, the shelf-life of parasite preparations was estimated by re-assaying at Days 0, 3, 7, and 14, since measurements from large clinical trials may take more than one day and it would be preferable to be able to use one parasite batch for such extended analyses.

### Assay controls

Parasites stained i) without primary Ab and ii) with serum from malaria naïve donors followed by the fluorescently labelled secondary antibody were used as negative controls. Positive control serum came from a pool of serum from malaria-exposed semi-immune adults living in Lambaréné, Gabon. As an additional positive control, infected RBCs were stained for HRP2 with a mouse monoclonal Ab (55A, anti-PfHRP2; Immunology Consultants Laboratories, Newberg, USA) at a 20 μg/ml concentration. Detection was performed using a 1/3,000 dilution of AlexaFluor 633 goat anti-mouse IgM (Invitrogen, Germany). Before analysing the cells with a flow cytometer, fluorescence microscopy was done to verify the effectiveness of the fluorescence stains and to verify the cellular localization of Ab-bound parasite proteins.

### Flow cytometry data acquisition and analysis

Parasite-infected cells were measured on a Becton Dickinson FACS Canto II flow cytometer equipped with the FACSDiva software version 6.1.2 (BD Biosciences, San Jose, USA) and an attached Carousel loader in high throughput mode. Relative fluorescence intensity of each event was analysed using FACSDiva software version 6.1.2 (BD Biosciences, San Jose, USA). Ab-reactivity was expressed as percentage of positive fluorescent cells (PPFC) and mean fluorescent intensity (MFI). Data acquisition was stopped after 50,000 events for each serum sample tested.

### Model-based analysis of flow cytometry data

Several model-based algorithms have been developed to automate the gating process thereby directly addressing several inherent limitations in gating-based analysis [19]. Some of these methods, including two popular model-based approaches, k-means [20] and an implementation of the Expectation Maximization algorithm (EM) [21] were tested on two experimental datasets. As part of this work, the Overlap Subtraction Algorithm (OSA) was developed and compared with model-based approaches. All described methods were benchmarked using manual gating as a gold standard. The OSA is implemented in the programming language R and is available from the authors.

### Design and mode of operation of the overlap subtraction algorithm

The algorithm effectively mimics manual gating whenever the gate is set with respect to an internal control. It detects overlapping areas of two datasets (e.g. between a control and the measurement of interest) in the two-dimensional space and sets a gate at the border of the overlap. Currently, the algorithm is able to process one colour staining, though it can be easily extended to process multicolour staining. The algorithm accepts files in the flow cytometry standard (FCS) 2.0 and 3.0 formats. MFI and PPFC are computed and reported as output.

With flow cytometry typically a fixed number of cells (e.g. 50,000) *C* are measured and analysed for each sample. Depending on the nature of the experiment, for each measured cell c_i_ ∈ *C* a vector of attributes a_1_…a_n_ can be assigned, e.g., colour intensities for different dyes, forward scatter (FSC), side scatter (SSC), etc. Generally, each cell is represented by a data point in the two-dimensional space, defined by the attributes a_1_ and a_2._

The algorithm starts by partitioning the whole value range for each attribute a_i_ of interest in β equidistant intervals, resulting in the vectors A_1_ and A_2_ of length β. The next step is to define two | A_1_ | x | A_2_ | matrices *T* and *C* for the test and control sample respectively. Then the values for *T*_ij_ and *C*_ij_ are calculated according to:

(1) $$\begin{array}{l} T_{\text{ij}}=\left|c\right|\geq A_{1i}\wedge \left|c\right|<A_{1i+1}+\left|c\right|\geq A_{2j}\wedge \left|c\right|<A_{2j+1}\\ C_{\text{ij}}=\left|c\right|\geq A_{1i}\wedge \left|c\right|<A_{1i+1}+\left|c\right|\geq A_{2j}\wedge \left|c\right|<A_{2j+1} \end{array}$$

Each entry in the matrices *T* and *C* stores the number of data points |c| whose values for the attributes a_1_ and a_2_ lie within a certain interval defined by the two vectors A_1_ and A_2_. Next, the percentage of data points coming from the test sample is determined according to the following formula:

(2) $$R_{\text{ij}}=T_{\text{ij}}/\left(C_{\text{ij}}+T_{\text{ij}}\right)$$

Following this calculation, positive entries are selected, i.e. entries in *R* that exceeds a certain threshold λ. To achieve a high specificity, λ is set to 0.99 by default, meaning that 99 percent of the data points that were counted for a particular entry come from the test sample. The correct gate is then set by finding the ω^th^ occurrence of an entry with:

(3) $${\text{Z}}_{\text{ij}}\leq \text{λ}$$

The parameter ω controls the sensitivity of the method. In practice it is used to fine-tune the gate’s distance to the negative control. By using low values of ω the gate is set close to the border of the negative control sample. Higher values of ω tend to produce gates that have a bigger gap from the control sample. After selection of relevant entries, the final gate is determined by Loess Regression through the selected coordinates.

### Statistical analysis of datasets from different populations

To detect differences in the MFI between groups due to vaccination, a linear regression model was used. To account for baseline differences on Day 0, it was included as covariate in the model (see Formula 4). Raw MFI measurements were log_10_ transformed before use in further analysis.

(4) $${\text{MFI}}_{\text{day}84}={\text{β}}_{0}{\text{+β}}_{1}\cdot {\text{MFI}}_{\text{day}0}+{\text{β}}_{2}\cdot \text{vaccine group}$$

For PPFC measurements, which cannot be assumed to follow a normal distribution, standard transformations to achieve normality as proposed by Ahrens *et al*. [22] did not work for both datasets. Therefore, log_2_ fold changes between Day 0 and Day 84 were calculated.

Between-group differences in the children dataset were tested by a one-way ANOVA followed by contrast extraction for comparisons of interest. Effects of vaccination within groups were tested by Student’s t-test.

Between-group comparisons and effects of vaccination in the adult dataset were tested using a non-parametric Wilcoxon test because even after transformation or calculation of ratios the data shows deviations from a normal distribution. To compare results derived manually as well as those obtained by automatic gating, Pearson’s correlation coefficients were calculated using log_10_ transformed Ab data measured as MFI. For PPFC comparisons Spearman’s rank correlation was used. Agreement between the methods was further evaluated with the Bland-Altman method [23]. The 95% confidence intervals for the mean difference are indicated for all Bland-Altman plots. All analyses were done with R v.2.13.0 [24] and statistical significance was defined as a two-sided p<0.05.

## Results

### Setup of assay parameters

**Figure 1:**
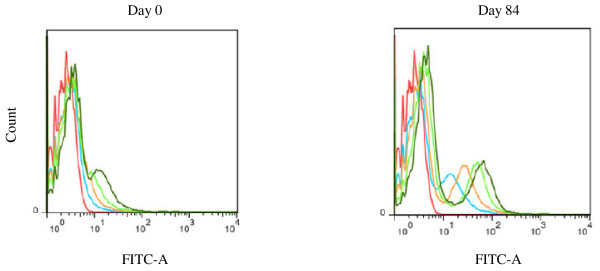
**Representative overlay showing the anti-plasmodial Ab responses of a semi-immune individual vaccinated with GMZ2. **The best separation between the negative and positive fluorescent cells is obtained when serum was diluted at 1/4,000. Test and control samples were treated as described in the methods. Note the increase in fluorescence intensity as shown by the shift to the right when parasites were incubated with serum diluted 1/32,000 (blue curve), 1/16,000 (orange curve), 1/8,000 (light green curve), 1/4,000 (green curve) or the control (red line) and the overall higher response after vaccination (Day 84).

To develop a standardized flow cytometric IFA to assess the Ab-reactivity to fixed *P*. *falciparum* parasites, a published fixation protocol [18] was adapted for use in flow cytometry. The basis for optimization was the best discrimination between positive and negative cells upon incubation with a serum pool from semi-immune individuals and preserved integrity and morphology of the cells. The final fixation and permeabilization conditions are given in the methods. Titration experiments showed that the use of semi-immune sera diluted at 1/4,000 followed by a 1/3,000 dilution of AlexaFluor 488 conjugated goat anti-human IgG best discriminated between negative and positive fluorescent cells (Figure 1).

### Assay validation procedure

**Figure 2:**
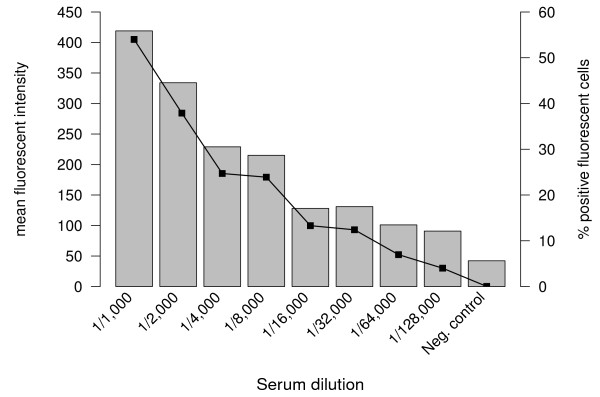
**Dose–response relationship in pooled serum. **Dilution series using a semi-immune serum pool. Bars show mean fluorescence intensity (MFI) and connected squares percentage of positive fluorescent cells (PPFC).

Following protocol development the new flow cytometry-based assay was validated using African semi-immune serum samples. These sera were selected on the basis of high anti-GMZ2 Ab-concentrations in ELISA. To assess concentration-dependent responses in antibody levels, a semi-immune serum pool diluted from 1/1,000 to 1/128,000 was used. Staining was specific (Figure 2) with only minimal cross-reaction to negative samples.

**Figure 3:**
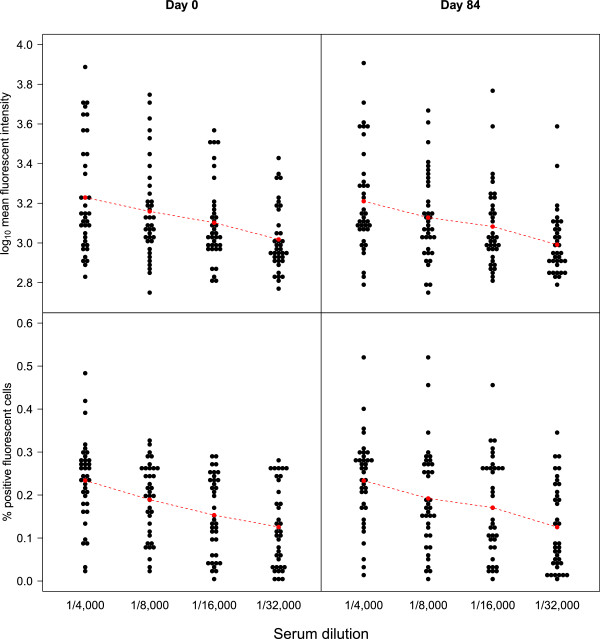
**Dose–response relationship in individual samples from semi-immune donors. **A set of 40 paired Day 0 (left panel) and Day 84 (right panel) sera from the same semi-immune population as in Figure 2. Dose-dependent responses can be seen for both the mean fluorescence intensity (MFI, upper panel) and percentage of positive fluorescent cell (PPFC, lower panel).

In addition, experiments were performed using a set of 40 Day 0 and Day 84 sera from the GMZ2 phase Ib trial in Gabonese adults serially diluted from 1/4,000 to 1/32,000. As expected, the PPFC and MFI values were dependent on the serum concentration (primary antibody) used in the assay and showed a consistent and obvious dose-dependent response relation on the different time points (Figure 3).

### Application of model-based algorithms in flow cytometry data analysis

**Figure 4:**
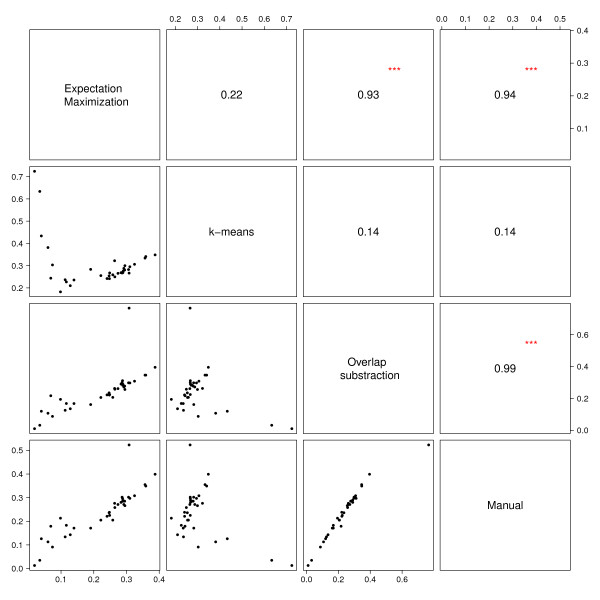
**Correlation matrix of results from different flow cytometry data analysis methods: adults. **PPFC measurements for Day 84 from Gabonese adults. The diagonal separates scatterplots (lower part) and the respective correlation coefficients (upper part).

**Figure 5:**
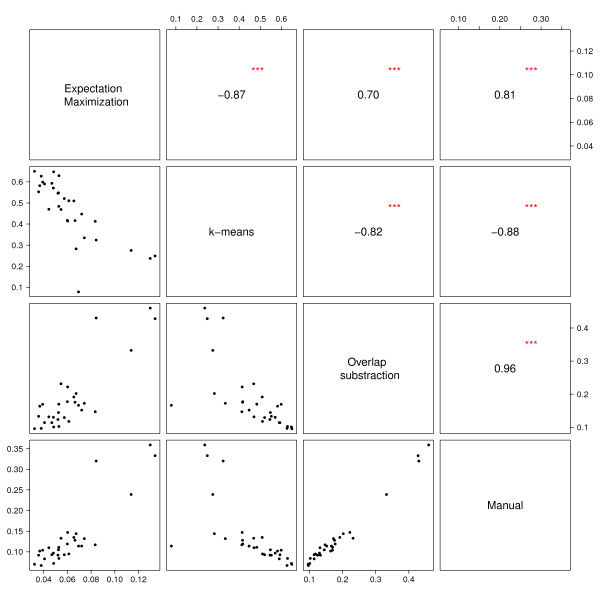
**Correlation matrix of results from different flow cytometry data analysis methods: children. **PPFC measurements for Day 84 from Gabonese children. The diagonal separates scatterplots (lower part) and the respective correlation coefficients (upper part).

**Figure 6:**
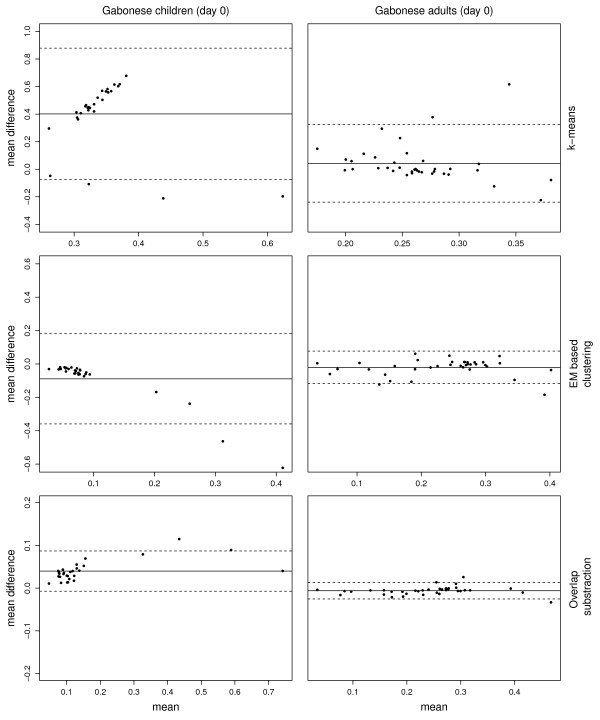
**Representative Bland-Altman plots obtained by comparing different analytical approaches. **The *x*-axis shows the mean of both computationally and manually derived estimates for the PPFC and the *y*-axis the difference between them. The inner solid line represents the mean difference for PPFC values while the outer dotted lines denote the lower and upper 95% limits of agreement between the different strategies.

Model-based gating algorithms were tested on two datasets. Of these, only two methods (k-means and the EM algorithm) tend to produce results that were comparable to those obtained by manual gating. They were selected and their performance was further evaluated in comparison to the manual gating strategy. Considering the MFI, results from the two methods do significantly correlate (p<0.001) with those obtained manually in both datasets. In contrast, k-means produced non-significant results for PPFC on Day 0 and 84 in the population of Gabonese adults when compared to manual gating. In the pediatric dataset, k-means-based results for PPFC measurements were even negatively correlated with those derived by manual gating (ρ=-0.93 on Day 0, ρ=-0.89 on Day 84, both p<0.001) (Table 1). Figures 4 and 5 show correlation matrices from Gabonese adults and children comparing the different analytical approaches using Day 0 PPFC measurements. Despite the significant correlation in most comparisons, Bland-Altman analyses show considerable lack of agreement between k-means, EM and manual gating for both, MFI and PPFC (Table 2). In both datasets k-means tends to under-estimate whereas EM over-estimates the MFI using results from the manual gating as reference. With regards to the PPFC among the children population, k-means over-estimates it by 40% and 34% on Day 0 and Day 84 respectively when compared to the manual gating. The poor performance of these methods on the datasets therefore motivated the development of a new method for data-driven gating. Since the different statistical approaches were not well-suited for the data, an algorithmic approach (OSA) was tested. In general, the algorithm produced results, which compared well (p<0.0001) to manually gated data (Table 1). In terms of MFI and PPFC for the different time points, the correlation appeared to be stronger for the adults (r ≥ 0.98) than for the children (r ≥ 0.79). In contrast to the other methods, OSA shows a high agreement with the results obtained from manual gating (Table 2). The expected absolute error for the PPFC in the semi-immune adults population is 30 and 60 times lower than for EM and k-means, respectively (Table 2). Figure 6 shows representative Bland–Altman plots with 95% limits of agreement (LOA). From all methods tested, OSA shows the smallest 95% LOA in terms of PPFC and MFI (Table 2).

**Table 1:** Correlation of the four strategies employed for gating raw flow cytometry data

**Manual gating**				
**Gabonese adults (n = 37)**^**a**^	MFI day 0	MFI day 84	PPFC day 0	PPFC day 84
k-means	r = 0.95	r = 0.89	ρ = 0.04^§^	ρ = 0.14^§^
	r^2^ = 0.91	r^2^ = 0.79		
EM*	r = 0.92	r = 0.89	ρ = 0.89	ρ = 0.94
	r^2^ = 0.85	r^2^ = 0.80		
Overlap subtraction	r = 0.99	r = 0.98	ρ = 0.99	ρ = 0.99
	r^2^ = 0.99	r^2^ = 0.96		
**Gabonese children (n = 28)**^**b**^	MFI day 0	MFI day 84	PPFC day 0	PPFC day 84
k-means	r = 0.71	r = 0.76	ρ = −0.93	ρ = −0.88
	r^2^ = 0.51	r^2^ = 0.59		
EM*	r = 0.61	r = 0.64	ρ = 0.78	ρ = 0.81
	r^2^ = 0.38	r^2^ = 0.41		
Overlap subtraction	r = 0.79	r = 0.83	ρ = 0.94	ρ = 0.96
	r^2^ = 0.62	r^2^ = 0.69		

*Expectation Maximization. r = Pearson’s correlation coefficient. r^2^ = Coefficient of determination. ρ = Spearman correlation coefficient. Ab (IgG) responses are expressed as mean fluorescence intensity (MFI) and percentage of positive fluorescent cell (PPFC). Correlations for MFI were calculated using log_10_ transformed data. ^a^Data excluded for 3 participants due to problems with data acquisition and inability of some algorithms to set an appropriate gate. ^b^Two participants have been excluded from analysis for the same reasons as above. All p-values are significant (p < 0.001) except for those marked with ^§^.

**Table 2:** **Bland**-**Altman analyses of the different data gating strategies**

	**Manual gating**			
**Gabonese adults (n = 37)**^**a**^	MFI day 0	MFI day 84	PPFC day 0	PPFC day 84
k-means	611	600.9	−0.04	−0.06
	(464.3, 757.7)	(350.5, 851.3)	(−0.09, 0.005)	(−0.12, -0.005)
Expectation	−388.3	−328.7	0.02	0.01
Maximization	(−600.2, -176.4)	(−604.8, -52.7)	(0.004, 0.04)	(−0.003, 0.03)
Overlap subtraction	−124.2	−98.1	0.006	−0.004
	(−160.3, -88.2)	(−143.4, -52.7)	(0.003, 0.009)	(−0.02, 0.001)
**Gabonese children (n = 28)**^**b**^	MFI day 0	MFI day 84	PPFC day 0	PPFC day 84
k-means	198.6	205.8	−0.4	−0.34
	(166.7, 230.6)	(176.8, 234.8)	(−0.49, -0.31)	(−0.42, -0.27)
Expectation	−208.9	−171.7	0.09	0.06
Maximization	(−290.4, -127.3)	(−214.5, -128.9)	(0.03, 0.14)	(0.05, 0.08)
Overlap subtraction	39.8	56.2	−0.04	−0.05
	(25.1, 54.5)	(43.6, 68.9)	(−0.03, -0.05)	(−0.06, -0.03)

Ab reactivity is expressed as mean fluorescence intensity (MFI) and percentage of positive fluorescent cell (PPFC). Data is given as mean differences of MFI and PPFC values (lower and upper 95% confidence interval) between the different approaches. ^a^Data excluded for 3 participants due to problems with data acquisition and inability of some algorithms to set an appropriate gate. ^b^Two participants have been excluded from analysis for the same reasons as above.

### Application of the cytometric IFA on sera from vaccinated subjects

**Figure 7:**
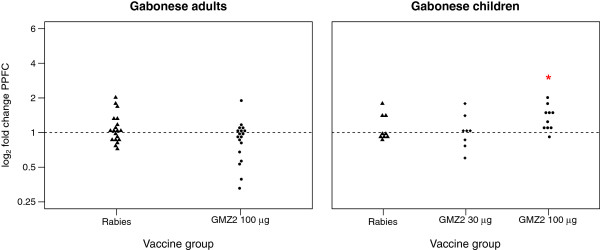
**Changes in Ab levels of Gabonese adults and children following immunization with GMZ2. **Data is expressed as log_2 _fold change in PPFC between Day 0 and Day 84. P-values were obtained by a one-way ANOVA and the Wilcoxon test for the children and adults’ data respectively.

The new method was applied to datasets from two GMZ2 phase Ib trials to detect possible effects of vaccination on Ab response. Each dataset consists of paired serum samples taken on Day 0 pre- and Day 84 post-vaccination. In total, 70 samples were analysed, 40 from semi-immune adults [8] and 30 from pre-school children [7], both from Gabon. Figure 7 illustrates the log_2_ fold changes in PPFC between Day 84 and Day 0 (baseline) responses of the different vaccine groups. Among children, most volunteers in the two subgroups vaccinated with GMZ2 had a higher response on Day 84 (63% and 90% who received 30 μg and 100 μg GMZ2, respectively). Out of all volunteers vaccinated with GMZ2, only those who received 100 μg GMZ2 showed a significant increase (p=0.003) in their Ab reactivity (1.33-fold, 95% CI: 1.15, 1.55), while no significant increase was observed in the 30 μg group (1.01-fold, 95% CI: 0.81, 1.27). Interestingly, 33% of all participants in the rabies-vaccinated group had also a higher response on Day 84. However, the remaining six showed no or minimal increase in reactivity on Day 84. As a consequence, no significant increase in vaccine response was detected on Day 84 (1.09-fold, 95% CI: 0.94, 1.28). In contrast to the pre-school children, no significant treatment effect on Day 84 was detectable neither in the 100 μg GMZ2 (0.83-fold, 95% CI: 0.71, 0.99) nor in the rabies control group (1.08-fold, 95% CI: 0.97, 1.21) of the adult volunteers. In addition, no differences between the vaccine groups could be detected in both datasets. Interestingly, by applying a linear regression model (Table 3) to the log_10_ transformed MFI values, which adjusts for the Ab reactivity on Day 0 (baseline), significantly higher vaccine responses (p=0.03) were detected in the 100 μg GMZ2 group compared to the rabies group. In the pre-school children population no significant between-groups differences were detected.

**Table 3:** Fold-changes in Ab reactivity after GMZ2 immunization of Gabonese adults and children

**Study populations**	**Mean (95% CI)**	**P-value**	**Comparison**
**Gabonese adults**	1.23 (1.02, 1.48)	0.03	GMZ2 100 μg/Rabies
**Gabonese children**	1.04 (0.92, 1.17)	0.52	GMZ2 30 μg/Rabies
	1.04 (0.93, 1.16)	0.48	GMZ2 100 μg/Rabies
	1.0 (0.89, 1.13)	0.98	GMZ2 30 μg/100μg

Ab reactivity is presented as mean fluorescent intensity (MFI). Data is shown as mean fold changes of the different comparisons (95% confidence interval). P-values for MFI comparisons derived by linear regression model.

## Discussion

A well-studied reaction of the immune system to malaria or vaccination with malaria vaccine candidates is the induction of antigen-specific antibodies [25]. Implementation of assays that adequately detect levels of antibodies induced by natural exposure or vaccination is critical for monitoring immunogenicity. In this respect, flow cytometric-based IFA techniques similar to the approach described here have extensively been employed to assess total IgG antibodies in the sera of humans infected with protozoan parasites different from *Plasmodium*[26-32]. With human malaria, some studies have adapted related techniques - mainly to analyse responses against plasmodial variant surface antigen [12,33-36], which may have a role in parasite virulence or be used as vaccine candidates.

Here, a novel approach for immunofluorescence assays, which incorporates flow cytometry and offers a rapid and reliable method of measuring total anti-plasmodial Ab in human serum, is presented. In contrast to conventional methods which utilize recombinant or synthetic peptides as antigen to assess Ab responses [9], the improved workflow has several advantages: i) *Plasmodium* parasites can be routinely maintained in continuous *in vitro* cultures to produce enough material for medium- to high-throughput assays; ii) the use of whole-cell preparations of *P*. *falciparum* may preserve the target protein’s antigenic properties better compared to soluble antigens [3], which could be essential for an effective anti-parasitic reaction to occur; and iii) the protein of interest is presented in its native context. Since fixed parasites remained intact and stable for more than 2 weeks when stored at 4°C, it is possible to analyse large sample numbers over an extended period of time. Furthermore, data acquisition using a flow cytometer equipped with a carousel or plate loader in high-throughput mode ensures rapid and consistent analysis of samples thereby reducing sample processing time and handling variations. This greatly improves the assay reliability when compared to the microscopic IFA technique, where the effort is limited by the microscopist’s experience and speed and where substantial variation among microscopists is common. The level of standardization and throughput that is possible using fully automated synthetic or recombinant peptides cannot be attained with such an approach.

The conventional method of manual gating of flow cytometry data is often investigator-dependent and difficult to standardize. To overcome these shortcomings several statistical methods have been proposed in the literature. After applying them to two study datasets, even the best performing ones (k-means and EM) showed high error rates when compared to expert manual gating. This disadvantage was remedied by the development of a new algorithm (OSA), which, in contrast to model-based methods, does not make any assumption on the data distribution and mimics manual gating strategies. OSA-derived results correlate well with those derived by manual gating. As a data-driven algorithm, OSA may not perform equally well in other experimental setups as it depends heavily on the data structure.

The whole workflow (cytometric IFA plus OSA) was validated using samples from two vaccine studies in malaria exposed adults and children who profoundly differ in their baseline anti-plasmodial immunity and showed a significant increase in specific Ab-reactivity against the GMZ2 vaccine after vaccination [7,8]. By applying the workflow, a moderate but significant increase in vaccine-induced Abs response was observed based on the PPFC, one month after a full immunization schedule (Day 84) in a subgroup of children who received the highest dose of GMZ2 (100 μg). Meanwhile, the effect induced by a lower dose of the vaccine (GMZ2 30 μg) was small and no significant treatment effect was detectable with this approach. A larger sample size may be required to detect a significant effect in this subgroup. In contrast to GMZ2-specific ELISA, which distinguishes GMZ2- from control-vaccinated children consistently, cytometric IFA results represent the integrated reactivity against all accessible parasite antigens after cell permeabilization. This decreases the ability to detect a specific signal but adds information about the size of the effect in the context of naturally acquired immunity and consequently complements antigen-specific methods.

Based on the PPFC outcome measure, no treatment effect was observed in semi-immune adults immunized with 100 μg GMZ2 (Figure 7). In contrast, a significant vaccination effect was detected between the two subgroups in the adult dataset when considering the MFI (Table 3). From the statistical point of view, a possible explanation for the contrasting observations in the two outcome measures (MFI and PPFC) may relate to the fact that outlying data points have a greater influence on MFI than PPFC. Consequently, PPFC is the more conservative measure and should be preferred in case of discordant results when no mechanistic explanation is present. In the present study, two different populations, which largely differ in their response pattern after vaccination were investigated. Children with no or very little immunity develop anti-plasmodial antibodies upon vaccination (increase in PPFC), whereas in semi-immune adults a vaccine-mediated boost of pre-existing anti-parasitic immune response that translates into improved parasite recognition (increased MFI) is expected. Therefore, the results are in line with the mechanistic concept of vaccination in naïve and pre-exposed populations, respectively.

The relatively high pre-vaccination antibody levels with specificities to different malaria parasite antigens reported in the adults population [8] contribute much to the large variation in the data. Therefore it is not surprising that a response to a single antigen is difficult to detect. Nevertheless, results from this investigation illustrate that a vaccine-induced increase in Ab- binding to fixed *Plasmodium* parasites is detectable by this methodology, demonstrating their potential functional properties [34]. However, the assay may need further adaptation for its use in subjects with no previous exposure to malaria and low immune responses as was observed in pilot experiments. IgG subclass-specific Ab responses, especially the cytophilic antibodies known to be associated with reduced risk of malaria [37,38], have not been addressed in the present study but can be integrated rather easily.

In summary, a new flow cytometry-based immunofluorescence assay is presented. It is a cheap, reliable and rapid method to detect and quantify anti-plasmodial antibodies in human sera and may be of value in malaria research. As a next step this workflow will be applied to samples from clinical phase II/III trials of malaria vaccine candidates to characterize Ab-mediated immune responses and identify correlates of vaccine-induced protection against malaria. The non-biased data-driven computational analysis tool (OSA) integrated in this methodology will be provided under a general public license to the scientific community.

## Abbreviations

RBCs: Red Blood Cells; Ab: Antibody; ELISA: Enzyme-linked immunosorbent assay; IFA: Immunofluorescent antibody assay; PBS: Phosphate buffered saline; HRP2: Histidine Rich Protein 2; EM: Expectation Maximization; MFI: Mean fluorescent intensity; PPFC: Percentage of positive fluorescent cells; OSA: Overlap subtraction algorithm; FSC: Forward scatter; SSC: Side scatter; FITC: Fluorescein isothiocyanate; LOA: Limits of agreement.

## Competing interests

The authors declare that they have no competing interests.

## Authors’ contributions

BM conceived the study and directed the experimental work. AA performed the experimental work shown in this paper. TE, BM and AA analysed the data. AA, TE and BM wrote the paper. ME collected samples and performed ELISA experiments. MT invented the GMZ2 vaccine and donated antigens for use in ELISA. SI contributed to the study design and reviewed the manuscript. All authors read and approved the final manuscript.
